# Speed versus accuracy instructions in the response time concealed information test

**DOI:** 10.1186/s41235-021-00352-8

**Published:** 2022-01-10

**Authors:** Till Lubczyk, Gáspár Lukács, Ulrich Ansorge

**Affiliations:** 1 Department of Cognition, Emotion, and Methods in Psychology, Faculty of Psychology, University of Vienna, Liebiggasse 5, 1010 Vienna, Austria; 2 Department of Philosophy, University of Vienna, Vienna, Austria; 3 Department of Psychology, Aoyama Gakuin University, Tokyo, Japan; 4 Vienna Cognitive Science Hub, University of Vienna, Vienna, Austria; 5 Research Platform Mediatized Lifeworlds, University of Vienna, Vienna, Austria; 6 Japan Society for the Promotion of Science, Tokyo, Japan

**Keywords:** Deception, Concealed information test, Response time, Speed–accuracy trade-off, Decision making

## Abstract

The response time concealed information test (RT-CIT) can reveal that a person recognizes a relevant item (probe) among other, irrelevant items, based on slower responding to the probe compared to the irrelevant items. Thereby, if this person is concealing knowledge about the relevance of this item (e.g., recognizing it as a murder weapon), this deception can be unveiled. In the present paper, we examined the impact of a speed versus accuracy instruction: Examinees (*N* = 235) were either presented with instructions emphasizing a focus on speed, with instructions emphasizing a focus on accuracy, or with no particular speed or accuracy instructions at all. We found that although participants responded to the probe and the irrelevants marginally faster when they had received instructions emphasizing speed, there was no significant difference between RTs of the different experimental groups and crucially no significant difference between the probe–irrelevant RT differences either. This means that such instructions are unlikely to benefit the RT-CIT, but it also suggests that related deliberate manipulation (focusing on speed on or accuracy) is unlikely to decrease the efficiency of the RT-CIT—contributing further evidence to the RT-CIT’s resistance to faking.

## Introduction

Undetected deception may lead to extreme costs in scenarios where optimal outcomes depend on rapid exchange of or the revelation of truthful information. These scenarios can include counterterrorism, pre-employment screening for intelligence agencies, or high-stakes criminal proceedings. Prior research has repeatedly shown that without special aid, based on their own best judgment only, people (including police officers, detectives, and professional judges) distinguish lies from truths on a level hardly better than mere chance (Bond & DePaulo, 2006; Hartwig & Bond, 2011; Kraut, 1980). For this reason, researchers have developed various deception detection methods, including the Concealed Information Test (CIT; Lykken, 1959; Meijer et al., 2014). In order to facilitate optimal applicability of the response time (RT)-based CIT and to potentially contribute with further insights regarding the underlying mechanisms of the examinee’s responses in the test, this study investigated the role of focusing on speed versus accuracy depending on corresponding instructions given to the examinees.

**Fig. 1:**
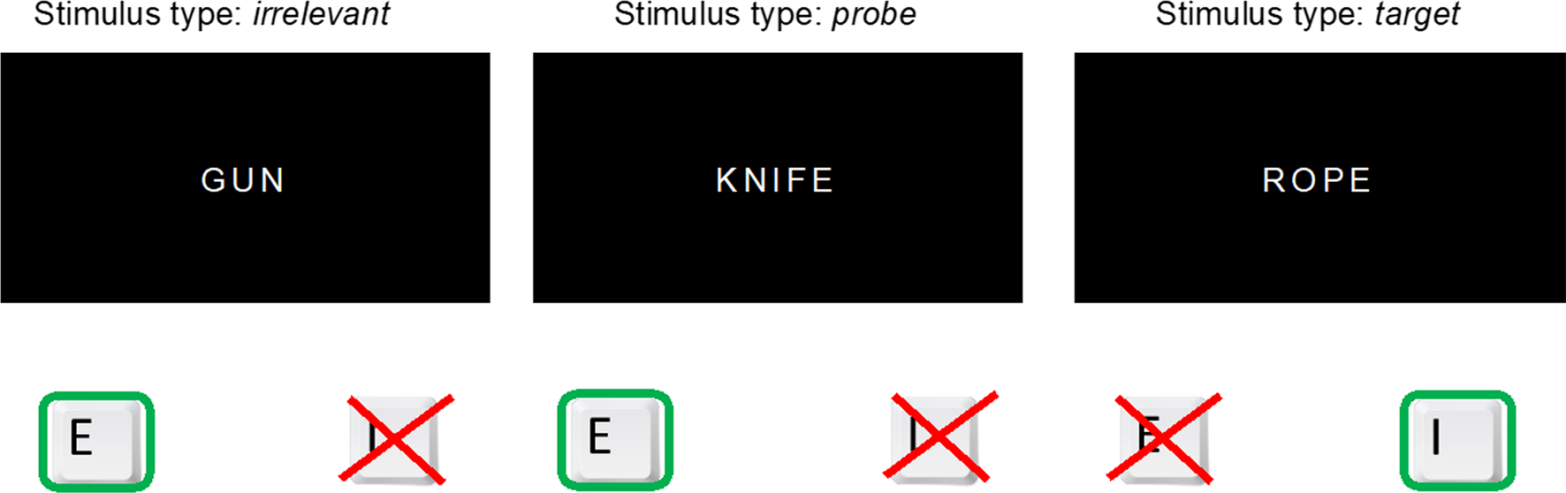
Item classification in the RT-CIT. *Note* Example items (as they would be displayed on a computer screen) and corresponding classification via keypresses for the Response-Time Concealed Information Test (RT-CIT), illustrated with a hypothetical murder case. The true murder weapon, in this case *knife*, serves as the probe, while four similar items, that are indistinguishable from the probe for naïve examinees, serve as irrelevants (e.g., here, one of these four is *gun*) and one additional irrelevant item serves as the target (here: *rope*). Irrelevants and probe have to be classified by the examinees with the same key (in this case, “E”), while the target requires a different response (in this case, “I”)

Generally, the CIT aims to disclose whether examinees recognize certain relevant items, such as a weapon used in a recent homicide, among a set of other objects, when examinees actually try to conceal any knowledge about the relevant item. In the RT-CIT, participants classify the presented items as the target or as one of several non-targets by pressing one of two keys (Seymour et al., 2000; Suchotzki et al., 2017; Varga et al., 2014). Typically, five non-targets are presented, among which one is the *probe*, which is an item that only a person familiar with the supposedly concealed information would recognize, and the rest are *irrelevants*, which are similar to the probe and, thus, indistinguishable from it for a person without knowledge. For example, in a murder case where the true murder weapon was a knife, the probe could be the word "knife," while irrelevants could be "gun," "rope," etc. Assuming that the innocent examinees are not informed about how the murder was committed, they would not know which of the items is the probe. The items are repeatedly shown in a random sequence, and all of them have to be responded to with the same response keys, except one arbitrary *target*—a randomly selected, originally also irrelevant item that has to be responded to with the other response key. Since knowledgeable examinees recognize the probe as the relevant item in respect of the deception detection scenario, it will become unique among the irrelevants and in this respect more similar to the rarely occurring target (Lukács & Ansorge, 2019). It is assumed that, due to this conflict between instructed response classification of probes as non-targets on the one hand, and the probe's uniqueness and, thus, greater similarity to the alternative response classification as potential targets on the other hand, the response to the probe will generally be slower in comparison with that to the irrelevants (Seymour & Schumacher, 2009). Consequently, based on the probe-to-irrelevant RT differences, knowledgeable (i.e., possibly guilty) examinees can be distinguished from naïve (i.e., innocent) examinees (Fig. 1).

A recent meta-analysis on RT-based deception detection included “speed instructions” as potential moderator for the effect size of probe versus irrelevant RTs: Authors found no statistically significant difference between studies that provided speed instructions (i.e., participants told to respond as fast as possible) and those who did not (*p* = 0.081; Suchotzki et al., 2017, p. 439). This indicates that there is no general large effect of focusing on speed in RT-based deception detection. Nonetheless, given the great heterogeneity among the examined studies and their methods, a number of other factors could have confounded this finding, and, in particular, these factors may affect the RT-CIT differently than the other methods included in the meta-analysis (see also Suchotzki et al., 2017, p. 444).

Within the present study, we manipulated not only speed instructions, but also, conversely, accuracy instructions (i.e., asking participants to make as many correct responses as possible). These two conditions were directly compared with a control group with no instructions at all regarding speed or accuracy.

Recent studies that were concerned with both speed and accuracy instructions have shown that these can indeed influence the results of perceptual decision-making tasks. Wenzlaff et al. (2011) showed that the mean RT difference between conditions of high and low levels of “sensory evidence” (easily versus hardly recognizable objects) was larger for participants that had received accuracy instructions than for those who were presented with speed instructions (74 ms vs. 37 ms; Wenzlaff et al., 2011, p. 1256). Similar results were presented by Herz et al. (2017), where RT differences between conditions of high coherence and low coherence in a moving dots task were larger with accuracy instructions than with speed instructions (Herz et al., 2017, p. 3). In both studies, accuracy rates in the tasks were less affected by the instruction change and remained in acceptable regions (e.g., 88 vs. 97 and 58 vs. 64% of correct responses for speed vs. accuracy instructions in conditions with high levels of sensory evidence and low levels of sensory evidence, respectively, Wenzlaff et al., 2011, p. 1256).

In the RT-CIT, for knowledgeable examinees, correctly categorizing the probe is generally more difficult than correctly categorizing the irrelevants. Hence, even though the RT-CIT may not be seen as completely analogous to the hard-to-discriminate stimuli in Wenzlaff et al. (2011) or to the conditions of low coherence as in Herz et al. (2017), this could serve as a potential indicator that, relative to speed instructions, accuracy instructions could increase the RT-CIT’s discrimination between knowledgeable and naïve examinees.

However, we could not rule out the opposite results: for example, it is possible that speed instructions increase cognitive load (Suchotzki et al., 2017), and there are a number of studies that indicate that cognitive load may increase probe–irrelevant RT differences (Lukács et al., 2017; Verschuere et al., 2015; Visu-Petra et al., 2013). Then again, as the particular CIT version used in the present study is already fairly demanding, further increments in cognitive load may have no additional benefit (Lukács et al., 2017).

### Speed–accuracy trade-off

The general notion that the accuracy of a response varies with the time taken to produce it has been studied in psychology for over a century (Heitz, 2014). As of today, it remains largely unpredictable if, for example, in a two-choice RT experiment, participants will focus more on giving the correct response or on responding fast. The speed–accuracy trade-off (SAT) continuum is conceived of as a variable threshold or criterion that triggers a decision once the criterion is passed by the participants’ collected information in favor of one of the options (Heitz, 2014). Depending on how liberal (being fast but relatively error-prone) or conservative (sacrificing speed for the sake of accuracy) the participant’s decision criterion (plus on how high or low their initial baseline level of information is), participants will reach said threshold more quickly or more slowly and consequentially make more or less informed decisions. As the sequential sampling of information is time-consuming and, hence, costly, participant’s best approach would be to sample as little information as possible to reach some specified compromise between certainty of the correct decision and time spent sampling (Wald, 1947). This accumulation of evidence toward one or the other decision option is considered to be a stochastic process as also observed in parallel neuronal processes (Heitz & Schall, 2012; Mansfield et al., 2011; van Maanen et al., 2011).

What makes a participant’s largely unpredictable SAT even more problematic for psychological studies and RT-based tests like the RT-CIT is that it can vary unpredictably not only across but also within participants, it can be adapted at will and between trials, and it sometimes even varies systematically by participants (Gueugneau et al., 2017; Liesefeld et al., 2015; Reuss et al., 2015; Wickelgren, 1977). The above-mentioned unpredictability of the participant’s SAT is often enhanced by conflicting instructions that simultaneously emphasize speed and accuracy (Heitz, 2014, p. 5). When, as is often the case in psychological studies (Wagenmakers et al., 2007), both speed and accuracy are considered as dependent variables in an experiment, the results are regularly influenced by a participant’s individual SAT.

This makes the SAT also relevant for the real-life applicability of the RT-CIT. When examinees take the RT-CIT, they are typically instructed to respond as fast as possible to the presented stimuli while also paying attention to continuously classify the items correctly as target or non-targets (e.g., Kleinberg & Verschuere, 2016; Verschuere et al., 2015). Unfortunately, conflicting as they may be, both speed and accuracy are necessary to some degree. If participants are given no time limit, they can fake the test by deliberately making their responses very slow to irrelevants (Suchotzki et al., 2021). If participants are allowed to make many mistakes, it would also allow them to simply not pay attention to the items in the task (e.g., just press random keys whenever a stimulus appears, without even identifying the stimulus first). Having a certain minimum rate of correct responses (as well as excluding incorrect responses from analysis) ensures that the participant paid attention to the items and, hence, their corresponding RTs are valid.

A simple and effective way to manipulate SAT in participants is to offer different sets of instructions. This is easy to implement, requires no additional training for participants, and yields large effects (Heitz, 2014; Howell & Kreidler, 1963). It has been observed that participants presented with conflicting instructions (speed and accuracy) tend to respond in a pattern more closely to participants that were presented with accuracy instructions (Howell & Kreidler, 1963). Therefore, we expected a similar pattern in our study as well: Since the RT-CIT to a degree requires fast as well as correct responses, we expected response patterns of participants receiving no specific instructions emphasizing speed or accuracy to more closely resemble those of participants with accuracy rather than speed instructions. This assumption is also in line with prior observations of generally low ERs to probes and irrelevants of the CIT: That is, in prior studies, participants with no instructions appear to have focused primarily on accuracy as opposed to speed.

In conclusion, we expected larger RT mean differences for accuracy instructions than for speed instructions and the control group to show results more similar to those accruing when participants are presented with accuracy instructions (rather than when they are presented with speed instructions). Given, however, the inherent demands of the RT-CIT for both speed and accuracy, it would also not be too surprising if related instructions had no substantial influence either way.

Finally, apart from the practical relevance of probe–irrelevant RT differences (whose increase implies higher classification accuracy), the results may also have theoretical implications. If slower responses to probes than to irrelevants predominantly reflect response conflict, we expect that the probe–irrelevant RT difference diminishes under speed instructions, giving way to a concomitant increase of the probe–irrelevant error rate (ER) difference, with more errors to probes than to irrelevants. However, if participants only apply more caution when responding to probes than to irrelevants, without substantial underlying response conflict, for instance, because participants only double-check their responses to the probes out of fear of doing something wrong (cf. Kuhl & Kazén, 1999), then the probe–irrelevant RT differences might decrease under speed instructions, enforcing a more liberal response criterion for all stimuli, but without a concomitant increase of a probe–irrelevant ER differences.

## Methods

### Participants

The experiment was conducted with voluntary participants recruited among undergraduate students in psychology at the University of Vienna, in exchange for “experimental participation” course credits. All participants had normal or corrected-to-normal vision and signed an informed consent (including agreement to publicly sharing their anonymous test data) before beginning the experiment. Each participant was randomly assigned to one of three conditions: *Control group* (no specific instructions regarding speed or accuracy), *Speed group* (instructions emphasizing speed), or *Accuracy group* (instructions emphasizing accuracy).

We initially opened 80 slots for participation in each of the three groups. We had registered to collect 25 more participants in each group, repeated up to a maximum of 130 participations (i.e., students who came and completed the test) per each group, if the Bayes factor (BF) for the one-way analysis of variance (ANOVA) across the three groups, for probe–irrelevant RT mean differences, had not reached 5.

For a power of 0.9 and alpha at 0.05, an effect size as low as Cohen’s *d* = 0.40 can be detected with 130 participants, in consideration of the critical between-subjects *t* tests comparing each two of the three groups (Champely, 2020). Assuming a base correct detection rate (CDR) of 0.80, an *SD* of 33.6 for “guilty” predictors (probe–irrelevant RT mean differences), an *SD* of 23.5 for “innocent” predictors, the CDR gain corresponding to the effect size of *d* = 0.40 would be 0.08 (hence, the improved CDR would be 0.88; see Lukács & Specker, 2020). In consideration of cost-efficiency, this potential improvement seemed a reasonable minimum size of interest to us (e.g., Lakens et al., 2018), especially in light of real-life cases’ differences likely being smaller than in strictly controlled laboratory experiments such as ours.

However, as can be seen in the results, the BF was well-above 5, at 19.45, already at 240 participants; hence, we stopped collecting at this point. We excluded five participants from the analysis based on our preregistered exclusion criteria (see below), leaving 235 valid tests in our analysis: 78 subjects (age = 22.1 ± 3.0; 26 male) in the accuracy group; 78 subjects (age = 21.9 ± 3.6; 27 male) in the speed group; 79 subjects (age = 22.5 ± 5.1; 24 male) in the control group).

### Procedure

At the beginning of the experiment, participants were asked to state and verify their given name, surname and birthday, as well as provide further demographic information. Participants were then presented with a random list of eight different dates (month and day) and eight different surnames that did not include their own and, regarding surnames, matched their surnames as closely as possible in character length. They were asked to select up to two dates and surnames of the list that in any way seemed familiar or meaningful to them or stood out to them from the rest of the list. Subsequently, five dates and surnames for the CIT were randomly selected from the non-chosen items (as this assured that the irrelevants were indeed irrelevant). One of these items was randomly chosen as the target, while the remaining four served as irrelevants.

In their respective condition (control vs. speed vs. accuracy), participants were randomly assigned to either completing the block with surname items first or to completing the one with date items first, with the respective other item category following in the second block.

The probe was the respective participant’s real surname as stated at the beginning of the experiment in one block and the participant’s birthday in the other block. During the RT-CIT, participants were asked to categorize items that were presented in the center of the screen by pressing either “E” or “I” on their keyboard. They were asked to press one of those keys, whenever they saw the probe or an irrelevant. Whenever the target appeared, they were asked to press the other key. Whether they were instructed to use “E” or “I” to categorize the probe and irrelevants and, respectively, the other key to categorize the target, varied randomly between subjects.

Apart from these main items (probe, target, irrelevants), we included two kinds of fillers: (a) expressions referring to familiarity and self-relatedness (e.g., “FAMILIAR,” “MINE,” etc.) that had to be categorized with the same key as the target (and, thus, opposite to the probe and the irrelevants), and (b) expressions referring to unfamiliarity and other-relatedness (e.g., “UNFAMILIAR,” “OTHER,” etc.) that had to be categorized with the same key as the probe and irrelevants. It is assumed (Lukács et al., 2017) that fillers further slow down responses to the probes because the probes have to be categorized together with the semantically incompatible expressions referring to unfamiliarity (cf. Nosek et al., 2007; Rosch et al., 1976). In addition, by increasing the complexity of the otherwise excessively simple task, fillers prevent strategically focusing on the target and, thereby, ignoring, to some extent, the probe and its meaning and relevance (Anderson, 1991; Hu et al., 2013; Reber, 1989; Verschuere et al., 2015; Visu-Petra et al., 2013). These assumptions, as well as the necessity of this specific arrangement of fillers, have been strongly supported by the findings of a recent study (Lukács & Ansorge, 2021).

The inter-trial interval (i.e., between the end of one trial and the beginning of the next) always randomly varied between 500 and 800 ms. In case of a correct response, the next trial followed. In case of an incorrect response or no response within the given time limit, the feedback “Falsch!” [“Wrong!”] or “Zu langsam!” [“Too slow!”] in red color appeared, respectively, in place of the stimulus for 500 ms, followed by the next trial.

To begin the test, participants were guided through three practice rounds. In the first round, they were asked to categorize only the filler items as being either familiarity-referring (“vertraut”, “mein”, relevant” [“familiar”, “mine”, “relevant”]) or unfamiliarity-referring (“unvertraut”, “fremd”, “unbekannt”, “andere”, “sonstiges”, “irrelevant” [“unfamiliar”, “foreign”, “unknown”, “other”, “other”, “irrelevant”]). In this first practice round, participants were required to have at least 80% valid responses. The time limit for their response was 1 s. If participants failed to reach 80% valid responses, they were reminded of the instructions and had to retake this practice round.

In the second practice round, participants were asked to categorize items as either unfamiliar (i.e., pressing the key assigned to classify the target) or familiar (i.e., pressing the key they were assigned to classify the probe and irrelevants). Depending on the condition, participants were either presented with dates or surnames when they were told to categorize the target as familiar and all other appearing items as unfamiliar. To secure that participants paid attention to the stimulus and that resulting differences in RTs (and ERs) in the main task were not caused by misunderstanding the instructions or uncertainty about how to respond, each trial in this round required a correct response. For this, participants were given an extended time limit for their response (10 s). To ensure that neither accuracy nor speed was already enforced in this practice round and to avoid bias in the following main task, each item was only shown once, and the round, thus, consisted of only six trials (probe + target + irrelevants). In the case of an incorrect response, participants were immediately reminded of the instructions and had to retake this practice round.

In the third and final practice round, fillers and main items were presented together and had to be classified as familiar or unfamiliar. The time limit for the participants’ response was decreased again (to the initial 1 s) and a certain rate of mistakes was allowed, though 60% valid responses for each item type (familiarity-referring filler, unfamiliarity-referring filler, target, main items [probe or irrelevants together]) were required to pass this round. Otherwise, participants were reminded of the instructions again and had to retake the practice round.

The main task followed and contained two blocks for each test. In the speed group, participants were instructed to respond as fast as possible to the items, react quickly to the items, and to focus on speed. They were then presented with all items, with the main items and probe consisting of either dates in the first block and surnames in the second block or vice versa, depending on random assignment. In order to avoid examinees regressing to their natural mean SAT (Heitz, 2014; Schouten & Bekker, 1967), they were reminded of their instructions between the first and second blocks. In the accuracy group, the procedure remained the same, with the exception that instead of the instructions with a focus on speed, participants were told to respond as accurately as possible, always press the correct response key and to focus on accuracy. In the control group, participants were not provided with particular speed or accuracy instructions. The response time limit remained at 1 s.

In each block, each probe, irrelevant, and target was repeated 18 times (hence, 18 probe, 72 irrelevant, and 18 target trials, in each block). The order of these items was randomized in groups: First, all six items (one probe, four irrelevants, and one target) in the given category were presented in a random order; then, the same six items were presented in another random order (but with the restriction that the first item in the next group was never the same as the last item in the previous group). Fillers were placed among these items in a random order, but with the restrictions that a filler trial was never followed by another filler trial and each of the nine fillers preceded each of the other items (probes, targets, and irrelevants) exactly one time. (Thus, 9 × 6 = 54 fillers were presented per block, and 54 out of the 108 other items were preceded by a filler.)

### Data analysis

We registered to exclude data from all participants, within each of the three experimental groups, with an accuracy rate further than three interquartile range [IQR] distance from the IQR, based on the IQR of each given group, for any of the following item types: (a) main items (probe and irrelevants merged), (b) targets, and (c) fillers (all fillers merged). Based on these criteria, five participants had to be excluded from the analysis. For all further analyses, responses below 150 ms were excluded.

All data analysis was carried out in R (R Core Team, 2019; via: Kelley, 2018; Lukács, 2021a; Morey & Rouder, 2018; Robin et al., 2011). For all one-way ANOVAs and all between-subject *t*-tests, we used Welch’s correction (Delacre et al., 2017, 2019).

## Results

Aggregated means for correct RT and error rate (ER), for each of the three experimental groups and the different item types, are displayed in Table 1.

**Table 1:** Mean correct reaction time (RT) and error rate (ER) per group

	Accuracy	Control	Speed
*RT*			
Irrelevant	487 ± 52	479 ± 49	470 ± 50
Non-target filler	562 ± 56	551 ± 50	550 ± 54
Probe	564 ± 59	555 ± 55	549 ± 55
Target	574 ± 54	570 ± 50	569 ± 55
Target filler	616 ± 61	614 ± 48	613 ± 51
Probe–irrelevant	76.4 ± 35.5	76.1 ± 36.5	79.1 ± 40.3
AUC	.969	.968	.957
*ER*			
Irrelevant	0.7 ± 0.9	0.6 ± 0.9	0.6 ± 0.8
Non-target filler	2.1 ± 2.1	1.7 ± 1.9	2.3 ± 2.5
Probe	4.8 ± 4.9	4.1 ± 4.7	4.5 ± 4.1
Target	15.3 ± 9.9	17.3 ± 11.4	18.7 ± 11.8
Target filler	19.7 ± 13.6	19.3 ± 11.6	20.9 ± 12.4
Probe—irrelevant	4.2 ± 4.8	3.5 ± 4.7	3.9 ± 4.2
AUC	.721	*.*692	.742

Means and SDs for RT means (in ms, valid trials only) and error rates (in percent), for different item types and per experimental group, as well as areas under the curve (AUC) for RT and ER probe–irrelevant differences as predictor variables

For the main question, whether the validity of the RT-CIT varies if participants are guided toward favoring either speed or accuracy over the other, the probe–irrelevant correct RT mean difference (probe mean RT minus irrelevant mean RT, per each participant, using all valid trials) was the dependent variable. As can already be seen in Table 1, the probe–irrelevant differences did not differ substantially between the groups. We conducted a one-way ANOVA across the three groups (speed vs. accuracy vs. control), and the results confirmed that there was no significant difference, *F*(2, 154.2) = 0.14, *p* = 0.866, *η*_p_^2^ = 0.001, 90% CI [0, 0.010], *η*_G_2 = 0.001, *BF*_01_ = 19.45. We still followed up with *t*-tests comparing each of the two groups in order to thoroughly assess pairwise group similarity with confidence intervals (CIs) and *BF*s. As expected, the *t*-test for accuracy versus control condition failed to reach significance, *t*(155.0) = 0.04, *p* = 0.968, *d* = 0.01, 95% CI [− 0.31, 0.32], *BF*_01_ = 5.81, as did the other group comparisons for speed versus accuracy instructions, *t*(151.7) = 0.46, *p* = 0.648, *d* = 0.07, 95% CI [− 0.24, 0.39], *BF*_01_ = 5.26, and for speed versus control condition, *t*(153.2) = 0.49, *p* = 0.625, *d* = 0.08, 95% CI [− 0.23, 0.39], *BF*_01_ = 5.20.

**Fig. 2:**
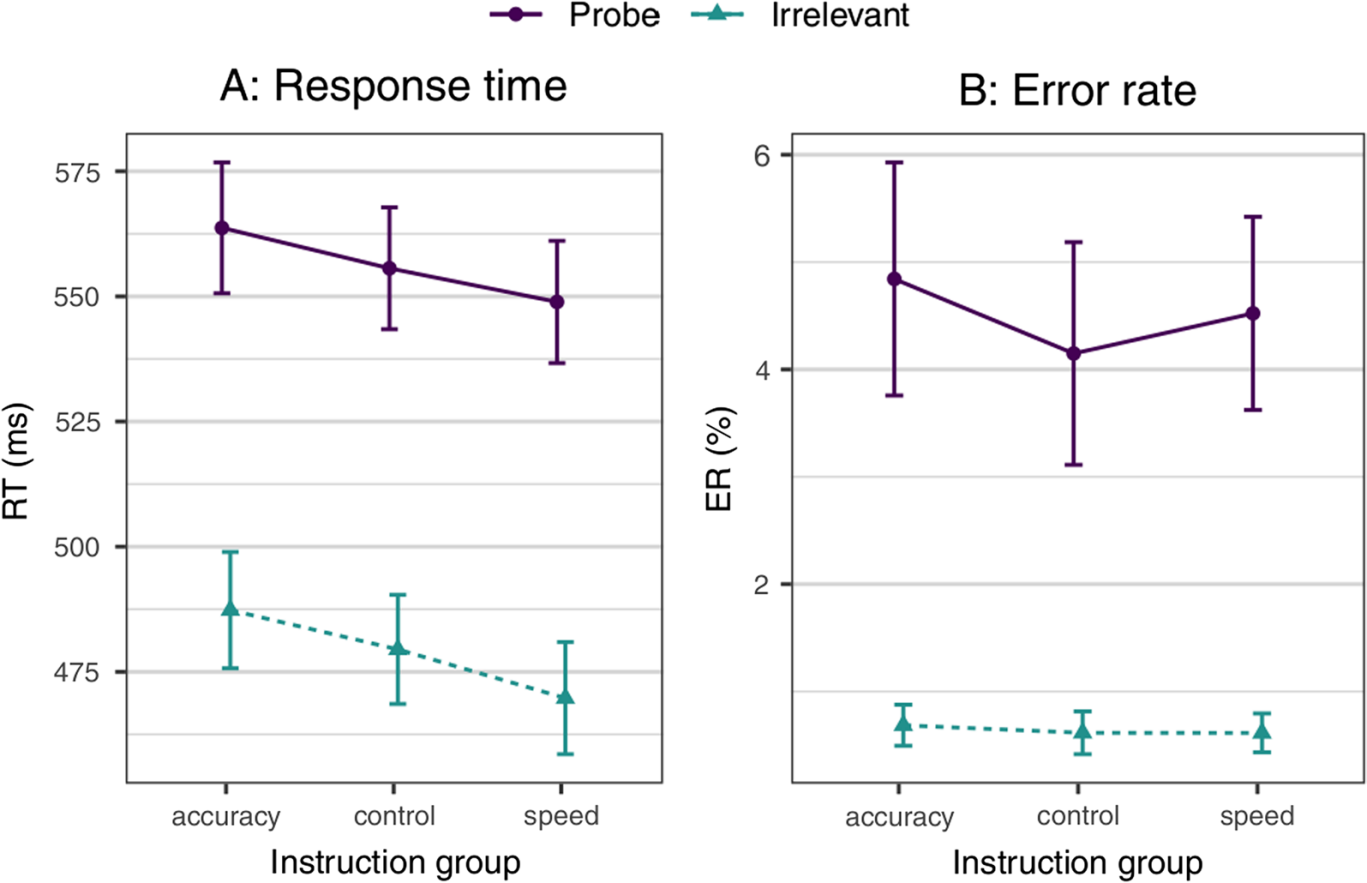
Analysis of variance (ANOVA), with the factors Stimulus and Instruction. *Note.* Mean and *SD*s for the response times (RTs; left panel **A**) and error rates (ERs; right panel **B**) of the probe and irrelevants across the three different instruction conditions (accuracy vs. control vs. speed)

As a supplementary analysis, we also conducted an ANOVA with the factors Stimulus (probe vs. irrelevants) and Instruction (speed vs. accuracy vs. no specific instructions) for said RT mean difference, see Fig. 2, left panel. The group-level results showed no significant difference, *F*(2, 232) = 2.02, *p* = 0.134, *η*_p_^2^ = 0.017, 90% CI [0, 0.049], *η*_G_^2^ = 0.015, *BF*_01_ = 1.71, neither did the stimulus–instruction interaction, *F*(2, 232) = 0.16, *p* = 0.856, *η*_p_^2^ = 0.001, 90% CI [0, 0.010], *η*_G_^2^ < 0.001, *BF*_01_ = 20.08 (stimulus main effect: *F*(1, 232) = 997.05, *p* < 0.001, *η*_p_^2^ = 0.811, 90% CI [0.778, 0.835], *η*_G_^2^ = 0.344, *BF*_10_ = 1.38 × 10^83^).

In order to assess how error rates were affected by instruction change, participant’s ERs were analyzed analogously. We thus conducted a one-way ANOVA for probe–irrelevant error rate differences across the three experimental groups, *F*(2, 154.0) = 0.34, *p* = 0.710, *η*_p_^2^ = 0.003, 90% CI [0, 0.018], *η*_G_^2^ = 0.003, *BF*_01_ = 16.06., and again conducted follow-up informative pairwise comparisons in order to approximate the certainty with CIs and *BF*s. For these comparisons, we report nonparametric t-tests (Wilcoxon signed-rank test and rank-based *BF*), because the assumption for normality was violated for the accuracy difference in all three groups (e.g., Shapiro–Wilk test, *p* < 0.001). There were no significant differences between accuracy instruction and control, *U* = 3346.00, *p* = 0.350, *d* = 0.13, 95% CI [− 0.18, 0.44], *BF*_01_ = 4.75, nor between accuracy instruction and speed instruction, *U* = 3083.50, *p* = 0.884, *d* = 0.06, 95% CI [− 0.26, 0.37], *BF*_01_ = 5.72, or speed instruction and control, *U* = 3346.50, *p* = 0.349, *d* = 0.08, 95% CI [− 0.23, 0.40], *BF*_01_ = 4.83.

Analogously to our analysis of the probe–irrelevant RT mean difference, we conducted an ANOVA, with the factors Stimulus (probe vs. irrelevant) and Instruction (speed vs. accuracy vs. no specific instructions) for error rates, see Fig. 2, right panel. It failed to reach significance at the instruction level, *F*(2, 232) = 0.52, *p* = 0.598, *η*_p_^2^ = 0.004, 90% CI [0, 0.022], *η*_G_^2^ = 0.002, *BF*_01_ = 21.33, as well as for the interaction, *F*(2, 232) = 0.37, *p* = 0.691, *η*_p_^2^ = 0.003, 90% CI [0, 0.018], η_G_^2^ = 0.002, *BF*_01_ = 15.51 (Stimulus main effect: *F*(1, 232) = 167.90, *p* < 0.001, *η*_p_^2^ = 0.420, 90% CI [0.342, 0.486], *η*_G_^2^ = 0.260, *BF*_10_ = 4.85 × 10^31).

To calculate illustrative areas under the curves (AUCs) for probe–irrelevant mean RT differences as predictors, we simulated control groups for the RT data using 1,000 normally distributed values with a mean of zero and an *SD* derived from the corresponding empirical data as *SD*_real_ × 0.5 + 7 ms (which has been shown to very closely approximate actual data; Lukács & Specker, 2020). These simulated AUCs for probe–irrelevant RT mean differences were 0.957, 95% CI [0.934, 0.980] for the speed instruction group, 0.969, 95% CI [0.949, 0.989] for the accuracy instruction group and 0.968, 95% CI [0.949, 0.988] for the control group without specific instructions regarding speed or accuracy. An analogous analysis was conducted on the basis of the probe–irrelevant ER differences (simulated with SD_real_) and resulted in simulated AUCs of 0.742, 95% CI [0.693, 0.791] for the speed instruction group, 0.721, 95% CI [0.670, 0.772] for the accuracy instruction group and 0.692, 95% CI [0.642, 0.742] for the control group. These AUCs indicate—similarly to the results of the ANOVAs above—hardly any differences between the three groups.

### Exploratory analysis

Since it can be seen in Table 1 that, in the speed group, participants were indeed faster to respond to items than in the other two Instruction groups—for example for the irrelevants—we also conducted a 5-by-3 ANOVA for item type and Instruction. In this way, we assessed whether overall RTs would differ between the groups—which they did not, *F*(2, 232) = 1.03, *p* = 0.359, *η*_p_^2^ = 0.009, 90% CI [0, 0.033], *η*_G_^2^ = 0.007, *BF*_01_ = 4.63. An ANOVA only comparing irrelevants across the three groups also failed to reach significance, *F*(2, 154.5) = 2.29, *p* = 0.105, *η*_p_^2^ = 0.020, 90% CI [0, 0.053], *η*_G_^2^ = 0.020, *BF*_01_ = 2.77, as did further exploratory analyses in which we analyzed the first 50 trials (*p* > 0.19) of all participants for differences in overall RTs between the groups. This was done to explore whether participants regressed to their natural SAT throughout the test, as the instructions were shown before the respective RT-CIT block, and thus, instruction effects could have been diminished through participants’ adjustment—but this seems not to be the case.

After we had collected 100 participants, we started a post-experiment manipulation check for the remaining 140 participants. Out of these, 90 were either in the accuracy or in the speed group and thus had received specific instructions emphasizing either a focus on accuracy or a focus on speed. When asked if they remembered seeing such specific instructions referring either to accuracy or speed, 81 of those 90 participants immediately recalled their specific accuracy or speed instructions correctly. The remaining nine were initially confused about the question or did not understand what was meant—however, after clarification about where in the experiment they would have seen such instructions (prior to the main experimental blocks), all of these nine participants could correctly recall their specific instructions. With 90% of participants immediately recalling correctly the instructions of their specific Instruction group, it is safe to assume that the lack of substantial differences in our main results is not due to participants’ lack of processing or understanding these specific instructions.

## Discussion

The main result of our study is straightforward: Instructions emphasizing a focus on speed or a focus on accuracy do not substantially impact the validity of the RT-CIT, nor do they alter the test results in any significant way. Although participants responded to the probe and the irrelevants marginally faster when they had received instructions emphasizing speed, there was no significant difference between RTs of the groups and crucially, no significant difference between the probe–irrelevant RT difference either.

This does not come completely unexpected, as the inherent demands of the RT-CIT already include, to a certain degree, speed constraints as well as accuracy constraints. For the test to have interpretable results, participants must give a certain number of correct responses and, simultaneously, be confronted with a time limit for each response. In our study, this time limit was set at 1 s. The rationale behind these requirements is that, without them, suspects who take the test could apply systematic strategies to obtain a favorable result: Without a time limit, participants could slow down their responses to irrelevants, diminishing the probe–irrelevant RT difference. Similarly, if there is no requirement for a minimum number of correct responses, participants can simply press any response key without paying attention to the items, rendering the test useless again. A recent study investigated the faking of the RT-CIT and confirmed that, indeed, the lack of a response time limit makes the test fakeable, whereas the implementation of such a deadline diminishes this faking effect (Suchotzki et al., 2021).

Our findings thus indicate that the inherent demands of the RT-CIT are too strong to be affected by differing instructions regarding a focus on speed or accuracy. One could even suggest that, in the RT-CIT, it is irrelevant how the task is exactly phrased, as the classification task and the time constraints will be prioritized by participants. This also relates to the finding that it does not seem to influence the results of the RT-CIT whether captions are displayed throughout the test that remind participants of the response key setting (Lukács & Ansorge, 2019). These captions which, for example, consisted of the phrase “Familiar to you?” at the top, “Familiar = E” on the left and “Unfamiliar = I” on the right side of the screen, with “E” and “I” referring to the corresponding response keys, altered neither the RTs nor the probe–irrelevant RT differences significantly.

Regarding the real-life applicability of the RT-CIT, we were not able to improve the validity of the test by increasing said probe–irrelevant RT difference and thus facilitate the interpretation of the results. However, our findings at least suggest that it is also unlikely that the test will be rendered less effective by the examinees’ deliberate focusing on either accuracy or on speed. From this perspective, the present study contributes to the accumulating evidence that (as long as response time limits are ensured) the RT-CIT cannot be effectively manipulated (Norman et al., 2020; Suchotzki et al., 2021).

Apart from this, our study reassures the usage of the probe–irrelevant RT difference as the decisive predictor for the test. The AUCs suggest a correct classification rate of approximately 96% throughout all Instruction groups, whereas using participants’ accuracy rate would only result in AUCs of around 71%, regardless of group.

Previous SAT studies have shown robust effects of speed or accuracy instructions alone (Heitz, 2014; Howell & Kreidler, 1963); we ourselves specifically took care to repeatedly emphasize the given speed or accuracy instructions, and indeed, all participants correctly recalled their specific instructions in this regard at the end of the task. Even so, we cannot rule out that our speed and accuracy instructions were not effective enough and that more motivated participants may make greater efforts, which in turn might actually significantly affect the RT-CIT outcomes. Future studies on this topic could therefore employ stronger (e.g., monetary) incentives to increase motivation.

Alternatively (or, e.g., if monetary incentives prove ineffective as well), to examine the SAT and the RT-CIT from the theoretical perspective, future research could try to shift the manipulation from the instruction level to a behavioral level. For example, instead of, or in addition to, presenting participants with different instructions regarding a focus on speed or accuracy, such a study could make use of different response time deadlines. The ideal response deadline for the RT-CIT is unknown (Suchotzki et al., 2021) and a study with a deadline of, for example, 800 ms in the speed group (cf., e.g., Lukács et al., 2017) and a deadline of, for example, 1500 ms in the accuracy group (cf., e.g., Suchotzki et al., 2021) seems reasonable. Additionally, in the accuracy group one could make use of an error feedback following incorrect trials, as used in our experiment, while in the speed group such a feedback could be omitted. Such a study could once again try to not only improve the applicability of the test but also analyze whether, as hypothesized in our Introduction, a decreasing probe–irrelevant RT difference in the speed condition would cause a concomitant increase of the probe–irrelevant ER difference and thus point to an underlying response conflict, or whether the prolonged response to probes compared to irrelevants were not caused by such a conflict (cf. Burle et al., 2013; Kuhl & Kazén, 1999).

### Participant dropout

Unrelated to the present research question, this study will likely be the first publication to report a laboratory-based use of the RT-CIT with fillers. In this respect, it is worth noting that not a single out of our 240 participants left the experiment (“dropped out”) for any reason. This unambiguously shows that the RT-CIT with fillers has no dropout-related limitations in its applicability, contrary to what some authors (Koller et al., 2020; Suchotzki et al., 2018) previously suggested (but without solid grounds, inferred from online experiments only; cf., Musch & Reips, 2000; Olson et al., 2020; Zhou & Fishbach, 2016; Wojciechowski & Lukács, 2021; see also Lukács, 2021b)—for more details regarding this presumed concern, see “Appendix”.

## Conclusions

Our study shows, above all, that instructions to focus on either accuracy or on speed during the RT-CIT do not modify substantially the key probe–irrelevant RT differences. This shows, on the one hand, that such instructions are unlikely to benefit this method, but, on the other hand, it also suggests that related deliberate manipulation (focusing on speed or on accuracy) is unlikely to decrease the efficiency of the RT-CIT—contributing further evidence to the RT-CIT’s resistance to faking (Norman et al., 2020; Suchotzki et al., 2021). More generally, these findings contribute further evidence that the RT-CIT is not influenced by relatively minor differences in task instructions (Lukács & Ansorge, 2019).

### Significance statement

The Concealed Information Test (CIT) allows assessing the recognition of concealed information. In situations in which undetected deception can result in significant individual or societal costs, special deception detection techniques like the CIT could be invaluable. In the response-time (RT)-based CIT, the concealing of information is detected by the time it takes examinees to correctly classify items they are presented with. It generally takes them longer to correctly categorize the item that they are concealing knowledge about (the *probe*) than to categorize other, irrelevant items (*irrelevants*). We aimed to foster the process of securing an optimal applicability of the RT-CIT by examining the role of the examinee’s focus on speed or accuracy depending on corresponding instructions. By improving the real-life applicability of the test, we aimed to further improve the RT-CIT and consequently refine the validity of its results when used in situations like high-stakes criminal proceedings or anti-terrorism measures.

## Data Availability

We will share all data (raw and aggregated), presentation script, and analysis script, via a public repository.

## Appendix

### Dropout rates when using the RT-CIT with fillers

In view of the reported dropout rates (proportion of participants leaving the experiment before finishing) as observed in the online study of Lukács et al. (2017), some authors (Koller et al., 2020; Suchotzki et al., 2018) contended that the RT-CIT with fillers has a notable limitation in this respect, as compared to other task designs—perhaps implying that the task is too difficult for some participants (even though it is hardly different from any other common dual task in psychological experiments, e.g., the Implicit Association Test; Nosek et al., 2007). However, such criticism misses the crucial fact that the observed dropout rates (e.g., 9–14% in case of Lukács et al., 2017) are completely normal for any sort of online study, and well within the expected range: Reviewing 27 online studies, Musch and Reips (2000) report that, on average, ca. 32% of the participants who access the first page of an online experiment do not proceed further and that the total dropout rate can be at times as high as 93%, depending on various factors. Similarly, Zhou and Fishbach (2016) found that 18 out of 88 their reviewed studies reported a dropout rate that exceeded 30%. The reason mainly boils down to the very low cost of starting the experiment (essentially a click of a button), and the consequent similarly low sunk costs when leaving it early—but see Zhou and Fishbach (2016, p. 495) for a more detailed explanation.

Thus, in respect of dropout rates in the RT-CIT, the only relevant difference between the papers by Lukács et al. and other RT-CIT papers in online settings is that the former do report dropout rates, while others do not.^1^ Now, in the present study, 240 participants completed the task in our behavioral laboratory and none “dropped out,” neither did anyone indicate any wish to cease the task at any point, nor did anyone complain of excessive difficulty. Together with analogous findings by Olson et al., (2020; see also Lukács, 2021b; Wojciechowski & Lukács, 2021), all this evidence (and neither reason nor any evidence to the contrary) decisively refutes any arguments for dropout-related practical limitations of using fillers.^2^
